# Current Review of Renal Autotransplantation in the UK

**DOI:** 10.1007/s11934-020-00986-z

**Published:** 2020-07-15

**Authors:** Georgios Vrakas, Mark Sullivan

**Affiliations:** 1 Oxford Transplant Centre, Churchill Hospital, Oxford University Hospitals NHS Foundation Trust, Old Road, Headington, Oxford, OX3 7LE UK; 2 Nuffield Department of Surgical Sciences, John Radcliffe Hospital, Headington, Oxford, OX3 9DU UK; 3 Department of Urology, Churchill Hospital, Oxford University Hospitals NHS Foundation Trust, Old Road, Headington, Oxford, OX3 7LE UK

**Keywords:** Autotransplantation, Kidney, Ex vivo tumour resection, Transplant, Renal cell carcinoma, United Kingdom

## Abstract

**Purpose of Review:**

Advances in preservation and transplantation techniques have made renal autotransplantation (RA) a modality that can be utilized in complex renovascular diseases (renal artery aneurysms), high ureteric injuries, chronic kidney pain, as well as conventionally unresectable renal tumours. In the current review, we present the Oxford experience, the only UK commissioned centre to perform RA for complex renal cell cancers, and review the published RA experience from other UK centres.

**Recent Findings:**

The evidence and literature generated from the RA experience in the UK are largely limited to case reports. The main indications reported for performing RAs include renovascular disease, ureteral pathology and prophylaxis from radiation.

**Summary:**

Renal autotransplantation is an option for a highly select group of patients. It has short-term and long-term complication rates comparable to those of other major operations. Extensive preoperative counselling in conjunction with multidisciplinary professionals is of utmost importance for informed decision making.

## Introduction

The pursuit of nephron preservation and avoidance of renal replacement therapy has led to the development of renal autotransplantation. Renal autotransplantation (RA) has become a modality that has found applications in various challenging renal disease scenarios.

The first renal autotransplant was performed in 1963 by Hardy JD et al. in Jackson, Mississippi [1], for a high ureteral injury during an aortic operation. Since then, the indications have been extended and renal autotransplantation can be utilized for renovascular diseases, chronic kidney pain, as well as conventionally unresectable renal tumours.

With respect to stage T1 renal tumours, the open partial nephrectomy (OPN) is still considered the gold standard treatment [2, 3]. However, there are cases where OPN, or even robotic partial nephrectomy, are not technically feasible due to the position and/or size of the tumour near the hilum and renal vessels. The conventional approach in these cases is to proceed with radical nephrectomy rendering patients with solitary kidneys or chronic kidney disease anephric or with limited renal reserve, respectively.

The Oxford RA service was first set up in 2005 and since 2012 has received national commissioning support from NHS England for renal autotransplantation post ex vivo resection of tumours (RART). In this paper, we present the 15-year Oxford RA and RART experience and review of the published UK experience on RA for benign disease.

## Methods

We reviewed the records of all patients who underwent RA and RART at Churchill Hospital, Oxford University Hospitals NHS Foundation Trust from 2005 to 2019. Demographic, clinical, operative and postoperative data were collected. Descriptive statistical analysis was performed. Complications were classified by the Clavien-Dindo classification system.

Graft failure was defined as the time from autotransplantation to nephrectomy or need for permanent dialysis, whichever was earlier. Patient survival was defined as the time from autotransplantation until death. Delayed graft function was defined as the need for supportive dialysis after transplantation. Primary non-function was defined as failure of the allograft to function. Graft function was measured at 3 months post autotransplantation with the estimated glomerular filtration rate (eGFR, ml/min/1.73 m^2^), which is calculated with the abbreviated modified diet in renal disease (MDRD) equation. The eGFR difference was calculated using the latest preoperative eGFR. Patient characteristics were reported using mean and SD for normally distributed continuous data and median and IQR for non-normally distributed continuous data. Life tables and Kaplan–Meier curves were used to compare graft and patient survival, which were censored at 10 years. The univariate log rank test (Mantel-Cox) was used to test differences in survival. All tests were two-sided and *p* values < .05 were deemed statistically significant. Analyses were performed using IBM SPSS Statistics version 25.

We conducted a Medline and PubMed search from 1935 to 2019 to identify all publications related to RA from centres in the United Kingdom. Only case reports were identified and included. Key words used for the search were “[kidney autotransplantation United Kingdom]”, “[renal autotransplantation United Kingdom]”, “[kidney AND autotransplantation United Kingdom]” and “[renal AND autotransplantation United Kingdom]”.

## Oxford Surgical Assessment/Technique

Given that RA is a long and challenging operation, all patients undergo extensive pre-operative work up to assess their suitability and fitness for RA. Imaging assessment involves CT angiogram for all cases to clarify vascular anatomy (especially the presence of multiple arteries) and additional CT chest, abdomen and pelvis for all cancer cases (staging). All cancer cases are discussed at the weekly urology multidisciplinary cancer meeting and are considered for either partial nephrectomy and/or autotransplantation.

Patients that are considered suitable for RA are then seen by a transplant surgeon and renal transplant physician for informed discussions regarding renal transplantation, as well as haemodialysis, either temporary or long-term. When delayed graft function is anticipated, external jugular tunnelled lines are placed preoperatively in all patients who will proceed with RA.

The kidney is removed by both open and laparoscopic techniques (surgeon dependent), following the principles of live donor nephrectomy. Warm ischemia time is kept as short as possible and the kidney is flushed with 1lt cold perfusion (Soltran, Baxter Healthcare) on the back table.

For cancer cases, bench surgery involves removing perinephric fat, maximizing vessel length and excision of the tumour with minimal loss of normal renal tissue. Vessels are reconstructed with 6–0 polypropylene and vascular stumps are secured with 6–0 and 5–0 polypropylene. Collecting system defects are closed or reconstructed with 5–0 polydioxanone sutures. Following reconstruction on the back table, propofol is infused into the renal artery, vein and ureter in order to allow better identification of arterial/venous and collecting system leaks, which are then repaired accordingly. The kidney defect is then closed with 3–0 polydioxanone sutures with or without a surgical coagulant depending on the size of the cavity created by the tumour excision.

For benign cases, bench surgery is more straightforward and only involves removing perinephric fat and maximizing vessel length.

Following the back table, the kidney is re-implanted using the external iliac vessels and the ureteric reimplantation is performed by the ureteroneocystostomy technique (Lich-Gregoir) over a JJ stent. Transitional cell cancer (TCC) cases underwent direct, single layer, renal pelvis to bladder anastomosis (pyelo-cystostomy).

## Oxford RA Series

Over the last 15 years, 50 patients underwent RA at the Oxford University Hospitals. Thirty-seven of these patients underwent RART for conventionally unresectable renal tumours. There were five patients that presented with ureteric TCC and underwent RA and pyelo-cystostomy following removal of the diseased ureter. Eight patients underwent RA for benign indications. Laparoscopic nephrectomy was performed in 12 cases. Patients have been followed up for a mean of 60 months. Median age of 66.5 years for our cohort is significantly higher than other previously published studies [4••, 5••].

**Fig. 1:**
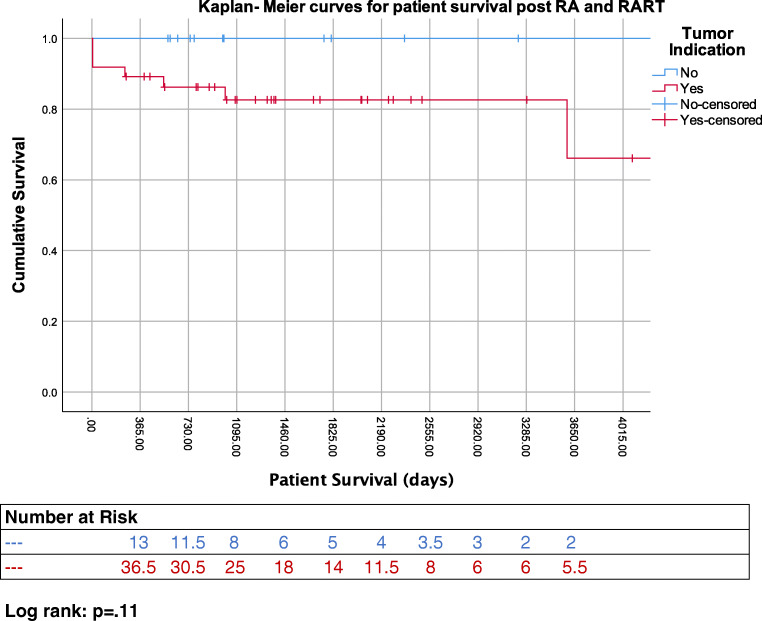
KM survival curves stratified by RCC vs non-RCC indication

Life tables estimate graft survival (censored) at 82%, 82%, 82% and 82% at 1, 3, 5 and 10 years, respectively. Patient survival was estimated at 92%, 87%, 87% and 75% at 1, 3, 5 and 10 years, respectively. Figure 1 depicts the Kaplan-Meier curve for patient survival post-RA for RCC vs non-RCC indication at our centre.

Demographics of all patients are shown in Table 1. Thirty-two percent of patients needed temporary haemodialysis during their early post-operative recovery. These were all RART cases. Five out of the 50 patients (10%) had failed graft/primary non-function and necessitated eventually long-term haemodialysis. Significant complications (Clavien-Dindo 3–5) were seen in almost half of the patients (46%). Urine leak was seen in eight cases and they were all RART cases (8/37, 21.6%). There were two pulmonary emboli cases that led to death (post-operative days 5 and 51). The autotransplant was removed in four cases (4/50, 8%): three cases of thrombosis and one case of ongoing pain symptoms. There were two 30-day mortalities, one due to post-operative day 2 aspiration and the second due to a post-operative day 5 fatal pulmonary embolus.

**Table 1:** Oxford patient demographics, clinical, intraoperative and postoperative variables

Median age, years (IQR)	66 (12.5)
Follow-up, months (mean, SD)	56.5 (46)
Solitary kidney	31/50 (62%)
Bilateral disease	16/50 (32%)
Primary disease	
1. Renal tumours	37
T3 RCC	21/37 (57%)
2. Transitional cell carcinoma	5
3. Benign conditions	
a. Ureteric stricture	4
b. Loin pain	1
c. Nutcracker syndrome	1
d. Cystic nephroma	1
e. Megaureter	1
Operation time, min (IQR)	415 (180)
Warm ischemia time, min (IQR)	4 (2)
Laparoscopic nephrectomy	12/50 (24%)
Temporary haemodialysis	16/50 (32%)
Long-term haemodialysis	5/50 (10%)
Complications (Clavien-Dindo III-V)	23/50 (46%)
eGFR decline, ml/min/1.73 m^2^ (IQR)	8 (23)
Length of stay, days	12.5 (13)
30-day mortality	2/50 (4%)

**Table 2:** Summary of critical literature for renal autotransplantation in the United Kingdom

Author	Year	Study design	Patient no.	Indication	Case
Hitchcock et al. [6•]	1993	Case report	1	Radiation	A 12-year-old with spinal Ewing’s sarcoma underwent renal sparing autotransplantation. Radiation nephritis was prevented by heterotopic transplantation of the kidney to a position outside the radiation field.
El Tayar et al. [7]	2003	Case report	1	Renal artery aneurysm	Report of a 2-cm-wide neck aneurysm that was treated by nephrectomy, ex vivo repair, and auto-transplantation.
Pretorian et al. [8]	2010	Case report	1	Reflux nephropathy	Patient underwent staged bilateral RA and was saved from renal failure
Chandak et al. [9]	2014	Case report	1	Mycotic aneurysm	In this case the patient developed a mycotic transplant renal artery patch aneurysm and was treated with surgical excision, in situ reconstruction, and subsequent allograft auto transplantation with successful outcome.
Maughan et al. [10]	2015	Case report	1	Renal artery aneurysms	This is a case of a 41-year-old primigravida who presented at 22-week gestation with severe abdominal pain, shock and foetal loss. A bleeding renal artery aneurysm was discovered at laparotomy and was radiologically coiled with sacrifice of the left kidney. Treatment of a contralateral aneurysm by autotransplantation of the right kidney allowed for preservation of residual renal function.

We present what is to our knowledge one of the largest and with the longest follow-up published series to date of patients undergoing RA, and most likely, the largest RART experience from a single centre. Oxford University Hospitals is the only centre in the UK that specializes in RART and is also performing RA for benign indications. The vast majority of the Oxford RA experience pertains to RART (37/50 cases, 74%).

Complications from these challenging and long operations were seen in almost half of our patients (23/50, 46%). Infectious complications were encountered in 13/50 cases (26%) and ranged from superficial wound infections to severe respiratory sepsis, requiring intensive care management. Renal vein thrombosis was diagnosed in three cases (3/50, 6%) and led to graft failure in two of these (2/50, 4%). Urine leaks were seen in 16% of our cases (8/50) and derived from the resected kidney parenchyma following RART. In our experience, the vast majority of urine leaks are treated conservatively by a 6F or larger JJ stent in the kidney, prolonged urethral catheterisation and keeping the drain, lateral to the reconstructed and re-implanted kidney, until the output dries out. Provided that the leaked urine is properly drained and not retained, the drain is sufficient to control the leak and allow the site to heal. Retaining the drain for potentially a significant period is part of the informed consent and preoperative consultation.

In contrast to previously published studies from non-UK centres [11], we observed a low local recurrence rate for RARTs (2/37, 5.4%). These local recurrences occurred at 1- and 4-year post-RART. Both cases that developed local recurrence had positive margins on the pathology report. An additional six pathology specimens were reported with positive margins; however, five of these patients have not developed local or distal recurrence after 2–4-year follow-up. The 6th case had their autotransplant removed after it had failed and no tumour was seen in the explant. We believe that these cases were reported as positive, due to our attempt to remain close to the tumour and save the hilar vessels. Figure 1 shows that patients that underwent RA, because of RCC (RART), had lower survival compared to the ones with a non-RCC indication; however, this was not statistically significant (82% vs. 100% 5-year survival, *p* = .11).

## UK RA Experience

Our literature review has showed published case reports with various RA indications from UK centres [6•, 7, –11] (Table 2). The first UK published RA study [6•] comes from St. George’s Hospital, London, and reports the case of a 12-year-old with spinal Ewing’s sarcoma, who underwent renal sparing autotransplantation. The plan was to treat the sarcoma with radiation and the team successfully prevented radiation nephritis by heterotopic transplantation of the kidney to a position outside the radiation field.

El Tayar et al. [7] from St. Mary’s Hospital, London, described the case of a 2-cm-wide neck aneurysm that was treated by nephrectomy, ex vivo repair and auto-transplantation. The team recommended digital subtraction angiography as the best diagnostic test and suggested surgical treatment of larger than 2-cm aneurysms in order to avoid hypertension and rupture of aneurysm.

Pretorian et al. [8], from Manchester, UK, presented a case where a young patient had significant impairment of health and quality of life due to recurrent urinary sepsis. This was a result of chronic bilateral urinary stasis which was leading to progressive renal impairment and would, eventually, cause end-stage renal failure. They performed staged (6-month interval) bilateral RA and managed to preserve her renal function and prevented her from progressing to end-stage renal failure. Timely intervention had meant that she has had no further admissions to hospital for urinary sepsis. The patient returned to full employment as a nurse.

In the case presented by Chandak et al. [9] from Guys and St Thomas’ Hospital, London, UK, the patient developed a mycotic transplant renal artery patch aneurysm, 6 months post-renal transplantation. The patient underwent right iliac fossa transplant nephrectomy and the aneurysm was removed by transecting the arteries high in the hilum. The reconstruction involved three renal arteries (two were pantalooned and one was anastomosed separately) and one short renal vein. The allograft was re-implanted with successful outcome into the left iliac fossa.

Maughan et al. [10] from The Royal London Hospital, London, UK, described a case of a 41-year-old primigravida who presented at 22-week gestation with severe, sudden onset, left-sided abdominal pain. The patient became soon haemodynamically unstable and was taken straight to the operating room, where the uterus was found to be displaced to the right by a large left retroperitoneal haematoma, which remained undisturbed. The foetus did not survive and was delivered by Caesarean section. A bleeding renal artery aneurysm was discovered at the CT that followed and was radiologically coiled with sacrifice of the left kidney. Treatment of a contralateral aneurysm by autotransplantation of the right kidney allowed for preservation of residual renal function.

## Conclusion

This UK RA review demonstrates that RA is a viable option in select patients for the treatment of complex renovascular diseases (renal artery aneurysms), high ureteric injuries, chronic kidney pain, as well as conventionally unresectable renal tumours with comparable outcomes. RA is a technically challenging operation with significant complications and a potentially long post-operative recovery. As such, it requires specialist surgical expertise and should be reserved as a last resort in carefully selected clinical scenarios and when conventional techniques have already been considered and excluded. Extensive preoperative counselling in conjunction with multidisciplinary professionals is of utmost importance for informed decision making.
